# Comparison of mcl-Poly(3-hydroxyalkanoates) synthesis by different *Pseudomonas putida* strains from crude glycerol: citrate accumulates at high titer under PHA-producing conditions

**DOI:** 10.1186/s12896-014-0110-z

**Published:** 2014-12-23

**Authors:** Ignacio Poblete-Castro, Danielle Binger, Rene Oehlert, Manfred Rohde

**Affiliations:** Facultad de Ciencias Biológicas, Center for Bioinformatic and Integrative Biology, Biosystems Engineering Laboratory, Universidad Andrés Bello, Santiago, 8340176 Chile; Helmholtz Centre for Infection Biology, Microbial Drugs Group, Braunschweig, D-38124 Germany; Helmholtz Centre for Infection Biology, Systems and Synthetic Biology, Braunschweig, D-38124 Germany; Helmholtz Centre for Infection Biology, Molecular Mechanism of Streptococci Group, Braunschweig, D-38124 Germany

**Keywords:** *Pseudomonas putida* strains, mcl-polyhydroxyalkanoates, Raw glycerol, PHA depolymerase, Metabolic engineering, Citrate

## Abstract

**Background:**

Achieving a sustainable society requires, among other things, the use of renewable feedstocks to replace chemicals obtained from petroleum-derived compounds. Crude glycerol synthesized inexpensively as a byproduct of biodiesel production is currently considered a waste product, which can potentially be converted into value-added compounds by bacterial fermentation. This study aimed at evaluating several characterized *P. putida* strains to produce medium-chain-length poly(3-hydroxyalkanoates) (mcl-PHA) using raw glycerol as the only carbon/energy source.

**Results:**

Among all tested strains*, P. putida* KT2440 most efficiently synthesized mcl-PHA under nitrogen-limiting conditions, amassing more than 34% of its cell dry weight as PHA. Disruption of the PHA depolymerase gene (*phaZ*) in *P. putida* KT2440 enhanced the biopolymer titer up to 47% PHA (%wt/wt). The low biomass and PHA titer found in the mutant strain and the wild-type strain KT2440 seems to be triggered by the high production of the side-product citrate during the fermentation process which shows a high yield of 0.6 g/g.

**Conclusions:**

Overall, this work demonstrates the importance of choosing an appropriate microbe for the synthesis of mcl-PHA from waste materials, and a close inspection of the cell metabolism in order to identify undesired compounds that diminish the availability of precursors in the synthesis of biopolymers such as polyhydroxyalkanoates. Future metabolic engineering works should focus on reducing the production of citrate in order to modulate resource allocation in the cell’s metabolism of *P. putida*, and finally increase the biopolymer production.

## Background

The consumption of non-renewable materials for industrial production of chemicals has given rise to environmental and energy concerns within society. Plastic products are considered essential materials to meet the needs of our current lifestyle and modern manufacturing. As an attempt to move towards a sustainable society, research has been undertaken to develop the microbial fermentation of renewable resources for the synthesis of biopolymers [1,2]. An example of the sustainable production of biopolymers is the large-scale production of polyhydroxyalkanoates (PHAs), which exhibit similar physical and mechanical properties to oil-based thermoplastics [1]. Bacteria have the capability to naturally accumulate PHAs as carbon/energy storage materials in their cytoplasmic space, usually when there is a nutrient imbalance in the environment: a high carbon concentration accompanied by the limitation of an inorganic nutrient [3]. Taking advantage of this evolutionary bacterial trait, commercialized PHAs are currently being produced by Metabolix (USA), Meridian Inc. (USA), Biocycle (Brazil), and Biomer (Germany), to name a few. They use sugars (cane or beet) and plant-based fatty acids as carbon feedstocks for the fermentation process in stirred-tank bioreactors, and obtain a diverse assortment of biopolymers. Despite the advances, one of the main drawbacks of PHAs for better market positioning is the high costs related to the production process [4,5]. To truly develop the synthesis of these polyesters and make them more cost-competitive against petroleum-based thermoplastics, it is crucial to find novel sustainable alternatives for large-scale production. Recently, production of PHAs using waste materials from agriculture residues and waste water treatment streams as a carbon substrate has shown to be a very promising alternative [6-8], since raw materials account for the majority of production costs in the bacterial synthesis of PHAs [4,9]. These processes have mainly focused on the synthesis of Poly(3-hydroxybutyrate) (PHB), the first and best-characterized PHA [1]. Despite the large range of applications possible with PHB and its co-polymer Poly(3-hydroxybutyrate-valerate) (PHBV), there is still an urgent demand for other types of biopolymers with unique physical and mechanical properties, with a particular emphasis on medical applications [10]. Medium chain length polyhydroxyalkanoates (mcl-PHAs) are polyesters synthesized mainly by gram-negative bacteria; most studies on their synthesis have focused on the use of Pseudomonas species as microbial cell factories [11-14]. By applying different metabolic engineering strategies [15-17] and fermentation conditions [18,19] the titer of this biopolymer was successfully improved, and it was demonstrated also that the monomer composition of the produced mcl-PHAs can be tuned [20-22]. In addition, given the high metabolic versatility shown by some *Pseudomonas putida* strains [23], waste materials from different streams have been used as carbon substrate to produce mcl-PHAs. Efficient synthesis of mcl-PHAs was obtained from animal wastes [24], PET [25] and raw glycerol [26]. Also, the conversion of biomass-derived compounds into mcl-PHA has been recently achieved [27,28]. One of the most promising raw materials for the synthesis of biobased chemicals is glycerol. The commercial price of this polyol has drastically decreased in the last decade due to the large industrial production of biodiesel from fatty acids, where glycerol is produced as a byproduct of the esterification process. For every 10 tons of biodiesel, 1 ton of glycerol is formed. Because of this, there is currently an oversupply of this commodity worldwide which has resulted in its classification as a waste product instead of a valuable good [29]. *P. putida* strains have been recently described to be capable of synthesizing mcl-PHAs from raw glycerol [25,26]. In this work, several *P. putida* strains were challenged to growth in raw glycerol as carbon and energy source and compared for their capability to produce mcl-PHA. The best PHA-producing *P. putida* strain was further engineered to enhance the synthesis of mcl-PHA in the cell. An exo-metabolome analysis was performed to identify and quantify byproduct formation during the fermentation process in bioreactors, and finally the causes for the low yield of biomass and biopolymers in *Pseudomonas putida* fed with glycerol were elucidated.

## Results and discussion

**Figure 1:**
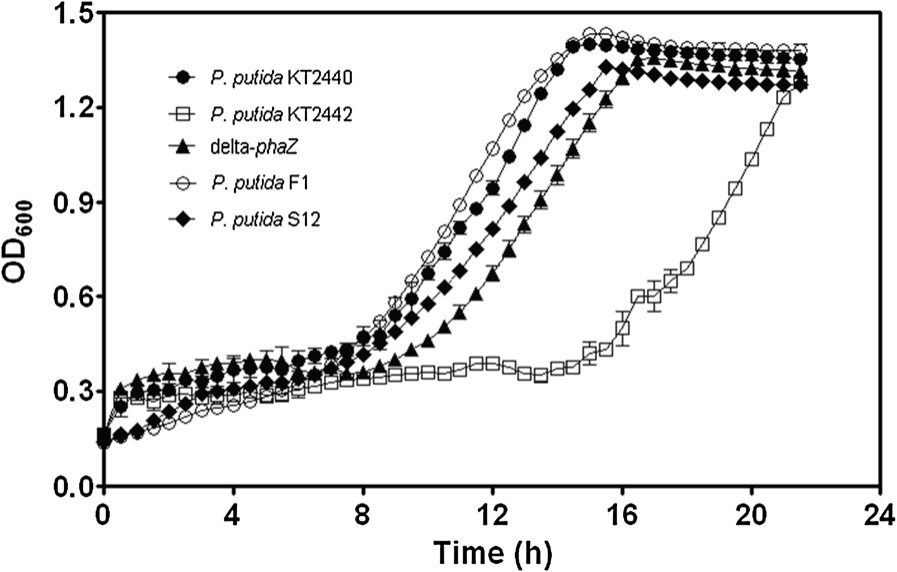
***P. putida*** **strains grown on raw glycerol (3 g/L) in batch cultures.** Values are the means of three independent biological experiments.

Raw glycerol is produced as a byproduct of the chemical conversion of fatty acids into biodiesel, a process that results in the following basic products: glycerol (60-80%), water (10%), organic matter (8%), ash (8%), and methanol (0.5-20%). Although methanol concentration seems to be minor, it exerts a high degree of toxicity to this feed stock material, thus limiting its use in industrial biotechnology. Bacteria from the genus *Pseudomonas* —especially *Pseudomonas putida*— possess a high metabolic versatility which allow them to colonize harsh and toxic environments [30]. In an attempt to test whether this raw material is suitable for the efficient production of medium chain length polyhydroxyalkanoates (mcl-PHAs), we selected three well-known PHA-producing strains: *Pseudomonas putida* KT2440, KT2442, and F1, and the solvent-tolerant *P. putida* S12 (Table 1). All of them have been previously reported to be able to grow in pure glycerol [12,31,32]. As shown in Figure 1, each *P. putida* strain grew well in minimal medium supplemented with 3 (g/L) of raw glycerol as carbon source. It is noteworthy to mention that we have not filtered or in any way modified the industrial byproduct glycerol. As already reported, *P. putida* KT2440 showed a long lag-phase (8 hours, this work) when grown on glycerol [33]. Escapa and co-workers found that by adding a small amount of octanoate or glucose, the length of the lag-phase can be reduced. In addition, elimination of the lag-phase was also achieved by knocking out the transcriptional regulator *glpR* (PP_1074). In this work, all tested *P. putida* strains have a similar lag-phase to that shown by KT2440, except for KT2442, which took more than 15 hours to begin the exponential growth phase (Figure 1).

**Table 1:** **Features of** ***Pseudomonas putida*** **strains used in this study**

**Strain**	**Genome size (bp)**	**GC content (%)**	**Isolated in**	**Solvent-tolerant**	**Biotechnological applications**
*P. putida* KT2440	6.181.863	61.5	Japan	No	Bioremediation Synthesis of biomaterials and chemicals
*P. putida* KT2442	N. K	N. K	Spontaneous rifampicin resistant	No	Bioremediation Synthesis of biomaterials and chemicals
*P. putida* F1	5.959.964	61.9	USA	No	Degradation of chloroaromatic compounds
*P. putida* S12	6.284.656	61.5	The Netherlands	Yes	Synthesis of added-value chemicals

N.K. Not known.

### Evaluation of PHA synthesis on different *Pseudomonas putida* strains in shake flask experiments

We next challenged *Pseudomonas putida* KT2440, KT2442, S12, and F1 to grow in minimal medium (M9) supplemented with 30 (g/L) of crude glycerol as the sole carbon source in batch cultures. After 72 hours of cultivation, cells were harvested so as to quantify the biomass and mcl-PHA content. *P. putida* KT2442, S12, and F1 were found to have a similar biomass concentration (Table 2). This was not the case for PHA production, where *P. putida* KT2442 produced twice as much biopolymer in comparison to the other two strains (Table 2). *P. putida* KT2440 was by far the most efficient PHA-producing organism among all tested strains, amassing 35% of its CDW as PHA, and achieving a biomass yield of 0.048 (g/g). This demonstrates the intrinsic metabolic differences between *P. putida* KT2440 and KT2442, which highly influence their PHA production capacity in the presence of PHA-unrelated carbon sources (glycerol, gluconate, and glucose). In this regard, a similar response has been found when both strains were compared in batch cultures using gluconate as carbon source; the production of mcl-PHA was 10-fold higher for KT2440 relative to KT2442 [34]. This phenomenon might be attributed to an inefficient transport capacity of 2-ketogluconate in *P. putida* KT2442, which would diminish the amount of available carbon within the cell, and negatively impact PHA synthesis. As glycerol is metabolized via a different metabolic route compared to the one used for gluconate, no gluconate-related oxidized compounds, e.g. 2-ketogluconate, are formed in the upper catabolic pathways. This may explain the reduced amount of synthesized biomass and mcl-PHA in *P. putida* KT2442.

**Table 2:** **Monomer composition of medium chain length PHA produced by different** ***P. putida*** **strains**

**Strain**	**CDW (g/L)**	**PHA (%wt)**	**PHA (g/L)**	**Monomer composition (%)**					
				**C6**	**C8**	**C10**	**C12:1**	**C12**	**C14:0**
***P. putida*** **KT2440**	4.23 ± 0.11	34.5	1.46 ± 0.21	0.83	16.2	74.6	2.9	6.0	0.59
***P. putida*** **KT2442**	3.45 ± 0.07	26.5	0.91 ± 0.13	t.d.	7.8	78.5	3.6	9.2	0.95
***P. putida*** **F1**	3.50 ± 0.14	10.3	0.36 ± 0.01	t.d.	14.4	71.3	3.9	11.4	t.d
***P. putida*** **S12**	3.20 ± 0.07	12.6	0.40 ± 0.05	t.d.	4.0	70.9	4.5	20.1	t.d.
***ΔphaZ***	4.20 ± 0.21	46.8	1.94 ± 0.17	1.1	16.3	72.3	3.4	6.9	t.d

The data were determined by GC/MS and are given as relative molar fraction (%) of C6: 3-hydroxyexanoate, C8: 3-hydroxyoctanoate, C10:3-hydroxydecanoate, C12: 3-hydroxydodecanoate, C12:1: 3-hydroxy-5-cis-dodecanoate, and C14: 3-hydroxytetradecanoate.

### Deletion of the *phaZ* gene in *P. putida* KT2440 enhances mcl-PHA synthesis using raw glycerol as a carbon and energy source

One of the first strategies for maintaining the titer of PHA inside of cells during the fermentation process is to remove the gene responsible for the depolymerization process of PHA (PHA depolymerase). However, the role of PhaZ proteins in the synthesis of mcl-PHA in *P. putida* strains is not well understood. In a previous work, disruption of the PHA depolymerase gene (PP_5004, *phaZ*) in *P. putida* KT2442 led to an increase in PHA synthesis using fatty acids as carbon source, but not when growing on glucose or gluconate [15], where the mutant strain produced less PHA in comparison to the wild-type strain. One could argue then, since glycerol is also a non-related PHA carbon source, it should show the same PHA-accumulation pattern as glucose or gluconate, resulting ultimately in no increase of the biopolymer. Nevertheless, in the study done by [15], they generated a *phaZ* mutant strain in KT2442, and as demonstrated in the research presented in this paper, this strain produces less mcl-PHA from no-alkanoate substrates. Therefore we generated a knockout mutant strain in the best PHA-producing strain, *P. putida* KT2440. The chromosomal deletion of *phaZ* (PP_5004) in *P. putida* KT2440 was performed as described in Methods. The Δ*phaZ* mutant strain was then subjected to PHA-production conditions in flask cultures in the same manner as described above with the wild type strain KT2440. There was no difference in total biomass yield (Table 2) between Δ*phaZ* and *P. putida* KT2440 wild type, however and most importantly, the first improved the PHA titer by 34% (Table 2). PHA depolymerase is believed to be required for the efficient production of the polyester —under PHA-accumulating conditions in batch culture— in *P. putida* KT2442 and *P. putida* U using fatty acids as the carbon substrate [35-37]. However, the opposite result has been reported for the same mutant strain (Δ*phaZ*) in *P. putida* KT2442 [15], and *P. putida* U [38]. Because we measured more mcl-PHA in Δ*phaZ* than its parent strain KT2440, we conclude that PHA depolymerase does not impose a negative effect on the PHA synthesis machinery. The PHA cluster genes orchestrates a simultaneous process of PHA synthesis and hydrolysis [35], making it impossible for the cell to degrade the PHA granule and further mobilize 3-hydroxyalkanoic acids for energy generation; this may be the primary reason for the greater accumulation of mcl-PHA in the Δ*phaZ* mutant strain when grown on glycerol.

### PHA morphology and monomer composition of PHA in *P. putida* strains grown on pure and raw glycerol

**Figure 2:**
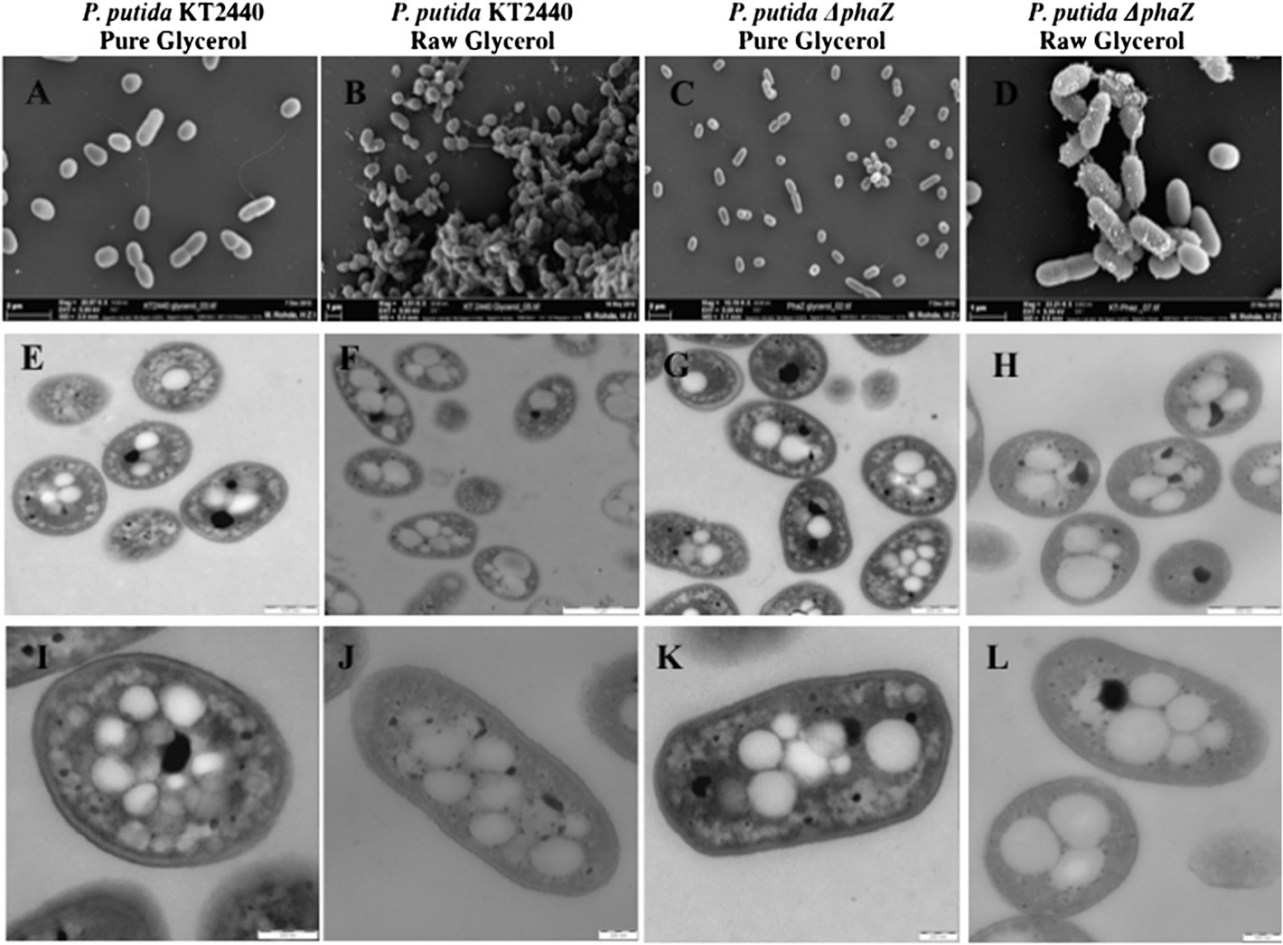
**Transmission electron micrographs of thin section of** ***P. putida*** **KT2440 (E,I, F,J) and Δ** ***phaZ*** **mutant strain (G,H,K,L).** Field emission scanning electron microscopy of *P. putida* KT2440 **(A,B)** and ∆*phaZ* mutant strain **(C,D)**.

As raw glycerol contains methanol and other toxic compounds, it may impose a stressful environment to cells. To get insight into the structural changes of cells and the accumulated PHA in both strains while growing in raw and pure glycerol, field emission scanning and transmission electron microscopy was applied. Micrographs were taken of cells at 72 h of cultivation, where maximum accumulation of PHA was obtained. Cells of the mutant and the wild-type strain did not show changes in their morphology when grown on raw or pure glycerol (Figure 2A,B,C,D), instead cells cultivated on crude glycerol stick together, where some of the impurities contained in the growing substrate can be observed (Figure 2B,D). The deletion of *phaZ gene* in *P. putida* KT2440 had no effect on PHA granules (Figure 2G,H). A different scenario was observed for PHA granules produced in both strains on raw glycerol, where the number of inclusion bodies was higher and more irregular in comparison to granules synthesized in cells cultivated on pure glycerol (Figure 2I,J,K,L).

**Figure 3:**
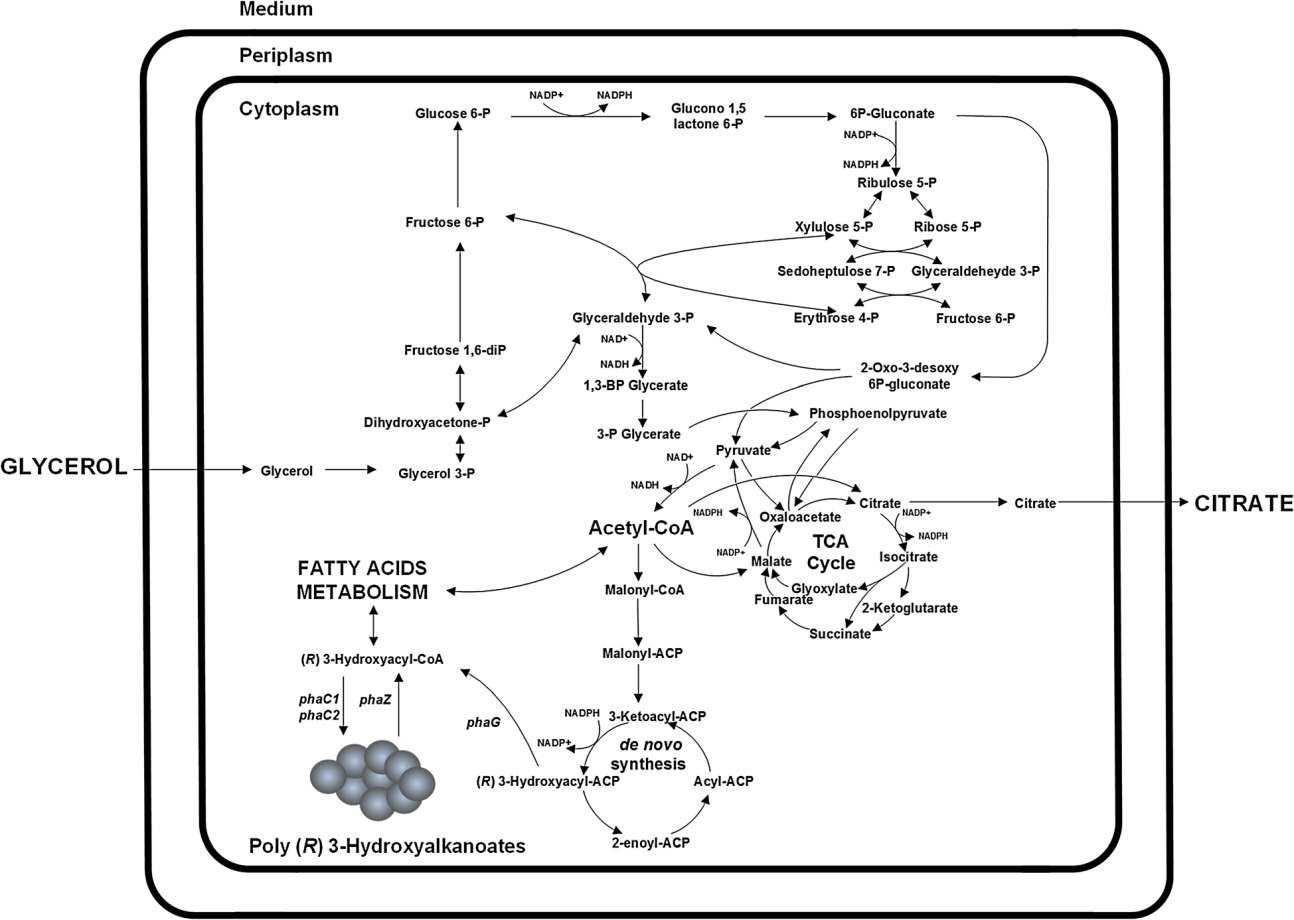
**Schematic representation of the metabolic reactions involved in PHA and citric acid production in** ***P. putida*** **KT2440.**

The dominant monomer characterized using GC-MS was 3-OH decanoic acid (C10) for all of the tested strains (Table 2). This has been shown to be true for various *P. putida* strains when they synthesize PHA from gluconate, glucose, or glycerol [12,16]. First, glycerol is converted in several steps to yield acetyl-CoA. Next, the glycerol is either used in the TCA cycle or used for the synthesis of *de novo* fatty acids (Figure 3). These *de novo* fatty acids are the precursor for PHA synthesis. Since 3-hydroxyacyl-ACP is restricted to 10 carbon substrates [39], a high content of 3-hydroxydecanoic acid is produced in the PHA polymerization cycle [16].

### Influence of aeration on the production of mcl-polyhydroxyalkanoates in *P. putida* Δ*phaZ* mutant strain

**Figure 4:**
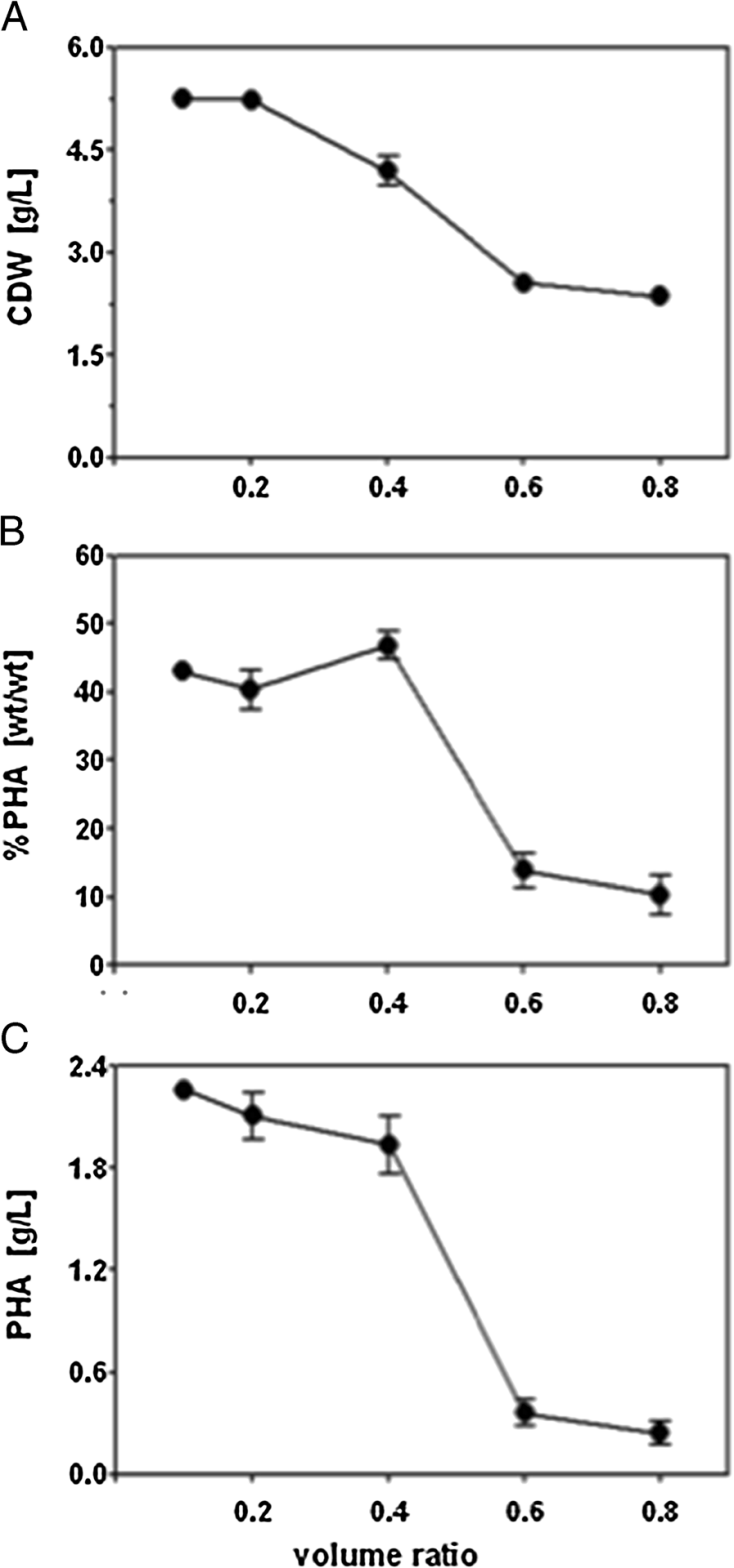
**CDW (A), %PHA (B), and PHA (C) content of Δ** ***phaZ*** **mutant strain grown on 30 g/L raw glycerol with different volume ration in flask batch experiments.** Values are the mean of three independent biological experiments.

We explored the influence of aeration on the synthesis of biomass and mcl-PHA in shaken-flask cultures of Δ*phaZ*, the mutant strain with the highest mcl-PHA production rates. Δ*phaZ* was grown in 500 mL flasks containing different volumes of minimal medium (50, 100, 200, 300, 400 mL, which correspond to a 0.1, 0.2, 0.4, 0.6, and 0.8 volume ratio, respectively) supplemented with 30 g/L raw glycerol. It was found that volume ratios higher than 0.2 exert a negative influence on biomass formation (Figure 4). In other words, as less oxygen is available with increasing the volume ratio, the oxidation state of the cell is disturbed, which resulted in inefficient energy generation, negatively impacting the production of biomass. PHA synthesis, in turn, also suffers from less oxic conditions, showing a 6-fold decrease of final PHA concentration (Figure 4). From this experiment we can conclude that PHA synthesis benefits from a well-aerated environment when raw glycerol is used as carbon source. This is crucial when developing an efficient PHA production process since biomass formation is critical to the production of biopolymers [19].

### Comparison of mcl-PHA synthesis by *P. putida* KT2440 and Δ*phaZ* mutant strain on mcl-PHA synthesis in well-controlled batch bioreactor

**Figure 5:**
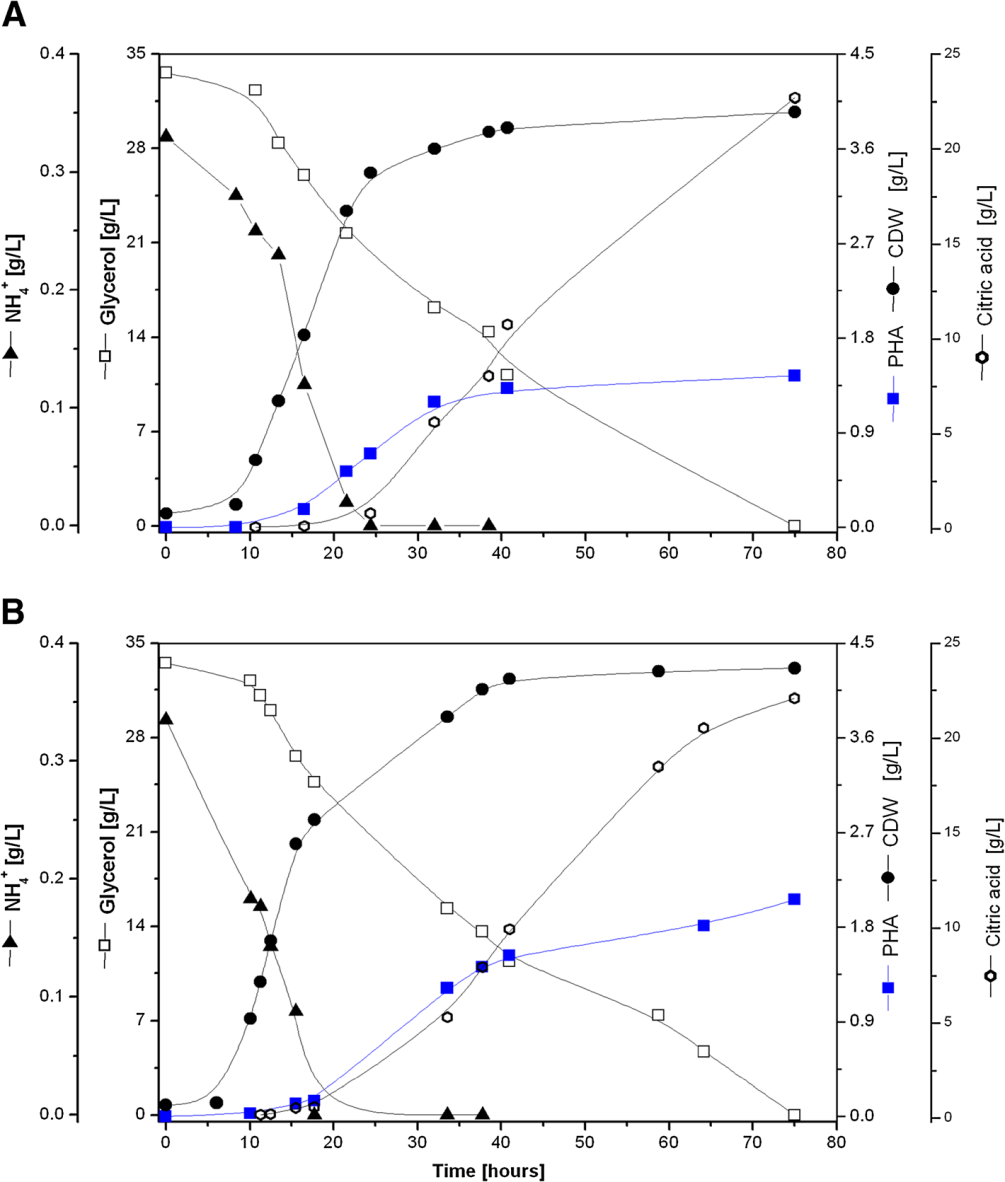
**Fermentation profile of** ***P. putida*** **KT2440 (A) and Δ** ***phaZ*** **mutant strain (B) in batch cultures using raw glycerol as carbon source (30 g/L).** Values are the means of two independent biological experiments.

To fully investigate the PHA production capacity of Δ*phaZ* and its parent strain KT2440, each biocatalyst was now cultivated in a more controlled environment using aerobic batch bioreactors. In addition, we did a detailed inspection of the exo-metabolome of the fermentation broth throughout the entire cultivation period via HPLC in order to characterize and quantify possible byproducts (citrate, formate, pyruvate, succinate, malate, acetate, see Methods section) produced by the cell. Both strains had an initial lag-phase of 7 h, after that time they started growing to a maximum specific growth rate of 0.22 h^−1^. Ammonium was below the detection limit at 18 h and 24 h for Δ*phaZ* and *P. putida* KT2440, respectively. As a consequence of nitrogen limitation, both strains began rapidly accumulating PHA until glycerol was completely consumed (Figure 5). Although Δ*phaZ* and *P. putida* KT2440 showed a similar total biomass yield when grown on glycerol (Table 2), Δ*phaZ* synthesized more PHA (~36% higher) in comparison to KT2440. It can be clearly seen in Figure 5 that Δ*phaZ* strain accumulates the biopolymer while consuming the available carbon source in the culture broth. Thus, the absence of this *loci* (PP_5004, *phaZ*) is not crucial, as previously believed, for the efficient synthesis of mcl-PHA in *Pseudomonas putida*. Another important finding was the detection of citric acid via HPLC, which reached a concentration of more than 20 g/L at the end of the cultivation period (Figure 5). It is important to mention that no other organic acid was detected in the broth culture during the fermentation process. Citrate has a final yield of 0.6 [g/g] for both the mutant and the wild type *P. putida* strain when grown on glycerol. This seems to be the principal reason for the observed biomass and PHA yield for strains grown on glycerol. Once glycerol is metabolized, the pool of acetyl-CoA replenishes both the TCA cycle and the *de novo* synthesis of fatty acids (Figure 3). It seems that more carbon was delivered to the TCA cycle since a higher amount of carbon was found in the form of citric acid than mcl-PHA. As methanol concentration in the culture broth is 0.188 g/L (see Methods section), this compound cannot be the principal factor influencing the final titer of citrate obtained at the end of the fermentation period (Figures 3, 5). A possible explanation of the high accumulation of citrate under the tested conditions is that PHA-producing environments (nitrogen limitation) increase the ratio of NAPH/NADP and/or NADH/NAD into the cell [40,41], which eventually results in repression of several enzymes belonging to the TCA cycle. We recently demonstrated that these cofactors play an important role in the synthesis of mcl-PHA in *P. putida* KT2440, where NADPH is consumed to increase the biopolymer production within the cell, resulting in a high level of NADH/NAD ratios [17]. One of the most affected proteins under high NADH levels is isocitrate dehydrogenase (PP_4012 and PP_4011), which catalyzes the conversion of isocitrate into oxalosuccinate. This effect limits the proper functioning of the Krebs cycle to further oxidize citrate. We have previously reported that intracellular concentrations of citrate were higher under elevated PHA levels in continuous cultivations using decanoate as carbon source, whereas both enzymes isocitrate dehydrogenase and lyase had low expression levels at the transcriptome and proteome level [13]. It is well documented that citric acid-producing microorganisms like the yeast *Yarrowia lipolytica* and the industrial mold *Aspergillus niger* (Table 3) accumulate large amount of citric acids (>150 g/L) and lipids under nitrogen limiting conditions [42-45]. As they do not posses the PHA machinery to naturally produce the polyester, they synthesize lipids instead, mainly in the form of triacylglycerol, reaching up to 50% of its CDW as lipids [46]. In yeast, N-limitation environments have been found to trigger the drop in the concentration of adenosine monophosphate (AMP) [47], thus inhibiting the isocitrate dehydrogenase leading to the accumulation of citrate and isocitrate [42]. As explained above, it seems that a similar mechanism drives the metabolic response of *P. putida* KT2440 under the PHA-producing conditions tested in this study. In order to fully confirm this hypothesis, further studies should aim at evaluating the intracellular flux distribution as well as the concentration of AMP, NADP/NADPH, and NAD/NADH ratios, in a more appropriate system such as continuous cultures (chemostat), where there are no changes in the specific growth rate or interferences by carbon catabolic repression [48], which affect the levels of the redox carrier compounds in the cell [49,50].

**Table 3:** **Citric acid-producing microorganisms using different carbon sources**

**Strain**	**Carbon substrate**	**Fermentation mode**	**Ycitrate/S (g/g)**	**Citrate (g/L)**	**Ref.**
***Y. lipolytica*** **mutant**	Rapseed Oil	Batch	1.5	175	[51]
***Y. lipolytica*** **A-101**	Raw Glycerol	Batch	0.6	112	[52]
***A. niger*** **mutant**	Beet, Cane Molasse	Batch	0.7	113.5	[53]
***A. niger***	Glucose	-	0.9	200	[54]
***P. putida*** **KT2440**	Raw Glycerol	Batch	0.6	20	This study
***P. putida ΔphaZ***	Raw Glycerol	Batch	0.6	20	This study

## Conclusions

This study shows that bacteria from the genus *Pseudomonas* can cope with the adverse conditions imposed by the chemical mixture of which raw glycerol from biodiesel production is composed. It also demonstrates the use of this cheap material in the efficient synthesis of mcl-PHA. Among all tested strains*, P. putida* KT2440 was the most efficient at mcl-PHA synthesis, amassing more than 34% of its CDW as PHA using raw glycerol as its only energy/carbon source. Disruption of the PHA depolymerase gene (*phaZ*) in *P. putida* KT2440 enhanced the biopolymer titer up to 47% (%PHA/wt). The low biomass and PHA titer found in *P. putida* KT2440 and the Δ*phaZ* strain on glycerol seems to be caused by the production of high levels of citrate during the fermentation process. Future metabolic engineering works should focus on eliminating or reducing the production of this organic acid in order to modulate resource allocation in the cell’s metabolism, and finally increase the specific volumetric productivity of PHA that is crucial for a cost-effective biopolymer production.

## Methods

### Bacterial strains

*P. putida* KT2440 (DSM 6125) and F1 (DSM 6899) were obtained from the German Collection of Microorganisms and Cell Culture (DSMZ, Braunschweig, Germany). *P. putida* KT2442 [55] and *P. putida* S12 [56] were obtained from CSIC Madrid (Spain) and RWTH Aachen (Germany), respectively.

### Construction of the *phaZ* knock-out mutant strain in *P. putida* KT2440

All PCR fragments generated for vector construction were subcloned into plasmid pCR®2.1 (Invitrogen, CA, USA), transformed into *E. coli* DH5α (Invitrogen, CA, USA), and validated by sequencing. Plasmid pEMG [57] was used for the construction of pEMG_ΔPP5004. Approximately 600 bp of the upstream and downstream regions of gene PP5004 were amplified using primers PP5004_UP_fw, PP5004_UP_rev, PP5004_DOWN_fw and PP5004_DOWN_rev (Table 4), Taq DNA polymerase (Qiagen, Venlo, The Netherlands) and genomic DNA from strain *P. putida* KT2440. Both PCR fragments were fused by PCR [58] and the resulting fusion PCR product was integrated into pEMG via *Xba*I-*Kpn*I restriction sites to generate pEMG_ΔPP5004. The resulting vector pEMG_ΔPP5004 was transformed into *E. coli* CC118λpir to generate donor cells for later tri-parental mating with *P. putida* KT2440 [55] and the helper strain *E. coli* HB101 as described by [59].

**Table 4:** **Primers used in this study**

**Primer**	**Sequence (5′-3′)**
PP5004_UP fw	TCTAGAGACATCCTGTTCTGGAAC
PP5004_UP_rev	CCCCTGTCAGGCCGCAGCTGGCACGTGACTCTTGGGTGAAGTAAAC
PP5004_DOWN_fw	CACCCAAGAGTCACGTGCCAGCTGCGGCCTGACAGGGGAAATGGATC
PP5004_DOWN rev	GGTACCGTTGAACAGCTCCTTGAC

To generate a single mutant of gene PP5004, genome editing was applied [57]. Therefore, vector pEMG_ΔPP5004 was co-integrated by a single crossover into the chromosome of *P. putida* KT2440 using tri-parental mating with E. coli HB101 as helper strain, as well as the donor strain E. coli CC118λpir pEMG_ΔPP5004. Successful homologous integration of the vector DNA and the successful genomic deletions was confirmed by PCR (data not shown).

### Cultivation conditions

#### Flask experiments

*P. putida* strains, kept as frozen stock in 25% glycerol at −80°C, were streaked on Luria Bertani agar plates and incubated for one day at 30°C. Single colonies were then picked from the plate and inoculated it into a 50 mL shake flask containing 10 ml of the above described medium (M9) and incubated overnight under aerobic conditions at 30°C and 180 rpm (Innova, Enfield, USA) set. By taking a calculated volume of the obtained cell suspension from the pre-culture (to begin the PHA-accumulating process with an initial OD of 0.05), 500 mL baffled shake flasks containing 100 mL of culture medium were inoculated and placed in a rotary shaker under aerobic conditions at 30°C. Each culture was carried out by triplicate. *P. putida* strains were grown in a defined M9 mineral medium consisting of (per liter) 12.8 g Na_2_HPO_4_^.^7H_2_O, 3 g KH_2_O_4_, 1 g NH_4_Cl, and 0.5 g NaCl. This basic solution was autoclaved and subsequently supplemented with 0.12 g of MgSO_4_^.^H_2_O, trace elements (mg/L): 6.0 FeSO_4_^.^7H_2_O, 2.7 CaCO_3_, 2.0 ZnSO_4_^.^H2O, 1.16 MnSO_4_^.^H_2_O, 0.37 CoSO_4_^.^7H_2_O, 0.33 CuSO_4_^.^5H_2_O, 0.08 H_3_BO_3_ (all filter-sterilized). 30 g/L of raw glycerol was then added to the medium which was previously autoclaved. The raw glycerol used in all experiments was obtained from the company Cremer Oleo (Hamburg, Germany). It contained 80% glycerol, 0.5% methanol, 10% ash, 3% organic matter, and 6.5% water.

#### Bioreactor fermentations

*P. putida strains* were grown in M9 medium supplemented with 30 g/L raw glycerol. Bioreactor batch fermentations were carried out in a 2 L top-bench BIOSTAT B1 bioreactor (Sartorius B Systems GmbH, Melsungen, Germany) with a working volume of 1.5 L, at 30°C. The aeration rate was set to 500 mL/Lmin using a mass flow controller (PR4000, MKS Instruments, Wilmington, MA, USA). The dissolved oxygen level was kept above 20% air saturation by control of the agitation speed up to a maximum of 700 rpm. The pH was maintained at 7.0 by automatic pH controlled addition of 0.5 M H_2_SO_4_ or 1 M of KOH.

### Analytical procedures

Cell growth was recorded as optical density (OD) at 600_nm_ (Ultraspec 2000, Hitachi, Japan). The cell dry weight was determined gravimetrically after collection of 10 mL culture broth for 10 min at 4°C and 9,000 × *g* (Eppendorf 5810 R, Hamburg, Germany) in pre-weighed tubes, including a washing step with distilled water, and drying of the obtained pellet at 100°C until constant weight. The ammonium concentration in cell-free supernatant was measured by a photometric test (LCK 303 kit, Hach Lange, Danaher, USA).

### HPLC analysis

The glycerol and organic acids concentration (citrate, isocitrate, succinate, fumarate, malate, pyruvate, and citrate) in cultivation supernatant was analyzed by HPLC Agilent 1260 (Agilent, Krefeld, Germany) equipped with an 8 mm Rezex ROA-organic acid H column (Phenomenex, USA) at 65°C with 0.013 N H_2_SO_4_ as the mobile phase (0.5 mL·min^−1^) followed by detection using a RID detector (Agilent serie1260).

### PHA characterization and quantification

PHA compositions of the polymer produced, as well as the cellular PHA content concentration were determined by gas chromatography (GC) and mass spectrometry (MS) of the methanolyzed polyester. First, 10 mL of the culture broth was placed in a falcon tube and centrifuged for 10 min at 4°C and 9,000 × *g* (Eppendorf 5810 R, Hamburg, Germany), following by a washing step with distilled water. The supernatant was poured away by pipetting and the cell pellet kept at −20°C for further process. Methanolysis was carried out by suspending 5–10 mg of lyophilized aliquots in 2 mL of chloroform and 2 mL of methanol containing 15% sulfuric acid and 0.5 mg/mL 3-methylbenzoic acid as internal standard, respectively, followed by incubation at 100°C for 4 h. After cooling, 1 mL of demineralized water was added and the organic phase containing the resulting methyl esters of monomers was analyzed by GC-MS. Analysis was performed in Varian GC-MS system 450GC/240MS ion trap mass spectrometer (Varian Inc., Agilent Technologies) and operated by the software MS Workstation 6.9.3 (Varian Inc., Agilent Technologies). An aliquot (1 mL) of the organic phase was injected into the gas chromatograph at a split ratio of 1:10. Separation of compounds of interest (i.e. the methyl esters of 3-hydroxyexanoate, 3-hydroxyoctanoate, 3-hydroxydecanoate, 3-hydroxydodecanoate, 3-hydroxy-5-cis-dodecanoate, 3-hydroxytetradecanoate) was achieved by a FactorFour VF-5 ms capillary column (30 m × 0.25 mm i.d. × 0.25 mm film thickness). Helium was used as carrier gas at a flow rate of 0.9 mL/min. The injector and transfer line temperature were 275°C and 300°C respectively. The oven temperature program was: initial temperature 40°C for 2 min, then from 40°C up 150°C at a rate of 5°C min^−1^ and finally up to 280°C at a rate of 10°C min^−1^. Positive ions were obtained using electron ionization at 70 eV and mass spectra were generated by scanning ions of *m/z* 50 to *m/z* 650. The PHA content (wt%) was defined as the percentage of the cell dry weight (CDW) represented by the polyhydroxyalkanoate.

### Transmission electron microscopy

Bacteria were fixed by chilling the cultures to 4°C and addition of glutaraldehyde (2%) and formaldehyde (5%). They were then washed with cacodylate buffer (0.01 mol l)1 cacodylate, 0.01 mol l)1 CaCl_2_, 0.01 mol l)1 MgCl_2_ 6H2O, 0.09 mol l)1 sucrose, pH 6/9) and stained with 1% aqueous osmium for 1 h at room temperature. Samples were then dehydrated with a graded series of acetone (10, 30, 50, 70, 90 and 100%) with incubation for 30 min at each concen- tration, except for the 70% acetone, which contained 2% uranyl acetate and was performed overnight. Samples were infiltrated with an epoxy resin, according to the Spurr for- mula for hard resin, for several days with pure resin. Ultrathin sections were cut with a diamond knife, counterstained with uranyl acetate and lead citrate and examined in a TEM910 transmission electron micro- scope (Carl Zeiss, Oberkochen, Germany) at an accelera- tion voltage of 80 kV. Images were taken at calibrated magnifications using a line replica. Images were recorded digitally with a Slow-Scan CCD-Camera (ProScan, 1024 × 1024, Scheuring, Germany) with ITEM-Software (Olympus Soft Imaging Solutions, Munster, Germany).

### Field emission scanning electron microscopy

Samples were fixed as above, washed with cacodylate buf- fer and then washed with TE-buffer (20 mmol l)1 TRIS, 1 mmol l)1 EDTA, pH 6.9). 50 ll of washed bacteria were applied to poly-l-lysine precoated cover slips (12 mm in diameter), which were left for 5 min, washed in TE-buffer, incubated with 2% glutaraldehyde in TE- buffer for 15 min and washed again with TE-buffer. Dehydration was carried out with a graded series of ace- tone (10, 30, 50, 70, 90, 100%) on ice for 15 min for each step, followed by 100% acetone at room temperature and critical-point drying with liquid CO_2_ (CPD 30; Bal-Tec, Balzers, Liechtenstein). Samples were then gold shadowed by sputter coating (SCD 500; Bal-Tec) and examined with a field emission scanning electron microscope Zeiss DSM 982 Gemini (Carl Zeiss, Oberkochen, Germany), using the Everhart Thornley SE detector and the inlens detector in a 50: 50 ratio at an acceleration voltage of 5 kV. Images were recorded onto a MO-disc. Contrast and brightness were adjusted with Adobe Photoshop CS3.
